# Sperm-borne microRNA-34c regulates maternal mRNA degradation and preimplantation embryonic development in mice

**DOI:** 10.1186/s12958-023-01089-3

**Published:** 2023-04-26

**Authors:** Long Cui, Li Fang, Lili Zhuang, Biwei Shi, Chao-Po Lin, Yinghui Ye

**Affiliations:** 1 Department of Reproductive Endocrinology, Women’s Hospital, Zhejiang University School of Medicine, Hangzhou, 310006 China; 2 Institute of Precision Medicine, Ninth People’s Hospital, Shanghai Jiao Tong University School of Medicine, Shanghai, 200041 China; 3 School of Life Science and Technology, ShanghaiTech University, Shanghai, 201210 China; 4 University of Chinese Academy of Sciences, Beijing, 100049 China

**Keywords:** microRNA, Sperm, *miR-34c*, Maternal mRNA degradation, Preimplantation embryonic development

## Abstract

**Background:**

Studies have shown that sperm-borne microRNAs (miRNAs) are involved in mammalian preimplantation embryonic development. In humans, spermatozoan *miR-34c* levels are correlated with in vitro fertilization outcomes, such as embryo quality and the clinical pregnancy and live birth rates. In rabbits and cows, *miR-34c* improves the developmental competence of embryos generated by somatic cell nuclear transfer. However, the mechanisms underlying the regulation of embryonic development by *miR-34c* remain unknown.

**Methods:**

Female C57BL/6 mice (6–8 weeks old) were superovulated, and pronucleated zygotes were collected and microinjected with an *miR-34c* inhibitor or a negative-control RNA. The embryonic development of the microinjected zygotes was evaluated, and the messenger RNA (mRNA) expression profiles of the embryos at the two-cell, four-cell and blastocyst stages (five embryos per group) were determined by RNA sequencing analysis. Gene expression levels were verified by reverse transcription–quantitative polymerase chain reaction. Cluster analysis and heat map visualization were performed to detect differentially expressed mRNAs. Pathway and process enrichment analyses were performed using ontology resources. Differentially expressed mRNAs were systematically analyzed using the Search Tool for the Retrieval of Interacting Genes/Proteins database to determine their biological functions.

**Results:**

Embryonic developmental potential was significantly reduced in zygotes microinjected with the *miR-34c* inhibitor compared with those microinjected with a negative-control RNA. Two-cell stage embryos microinjected with an *miR-34c* inhibitor presented altered transcriptomic profiles, with upregulated expression of maternal *miR-34c* target mRNAs and classical maternal mRNAs. Differentially expressed transcripts were mainly of genes associated with lipid metabolism and cellular membrane function at the two-cell stage, with cell-cycle phase transition and energy metabolism at the four-cell stage; and with vesicle organization, lipid biosynthetic process and endomembrane system organization at the blastocyst stage. We also showed that genes related to preimplantation embryonic development, including *Alkbh4*, *Sp1*, *Mapk14*, *Sin3a*, *Sdc1* and *Laptm4b*, were significantly downregulated after microinjection of an *miR-34c* inhibitor.

**Conclusions:**

Sperm-borne *miR-34c* may regulate preimplantation embryonic development by affecting multiple biological processes, such as maternal mRNA degradation, cellular metabolism, cell proliferation and blastocyst implantation. Our data demonstrate the importance of sperm-derived miRNAs in the development of preimplantation embryos.

**Supplementary Information:**

The online version contains supplementary material available at 10.1186/s12958-023-01089-3.

## Introduction

Preimplantation embryonic development is thought to be guided by regulatory factors present in the cytoplasm during oocyte growth. It was suggested that spermatozoa with a low RNA content (10–400 fg per sperm cell) provide only a paternal genome for fertilization and may not play a key role in early embryonic development [1]. However, upon fertilization, spermatozoa deliver a unique set of RNAs to oocytes, consisting of transfer RNAs (tRNAs), ribosomal RNAs (rRNAs), messenger RNAs (mRNAs) and noncoding RNAs (ncRNAs) [2]. Sperm-borne ncRNAs, including microRNAs (miRNAs), P*-*element*-*induced wimpy testis-interacting RNAs, tRNA-derived small RNAs and rRNA-derived small RNAs [3], may play distinct roles in early embryonic development [4] and epigenetic inheritance [5, 6].

MiRNAs function as post-transcriptional regulators by targeting mRNAs for degradation and/or translational repression [7]. Compared with embryos derived from sperm with a full complement of miRNAs, embryos derived from sperm partially deficient in miRNAs display a significant reduction in developmental potential, which can be rescued by microinjecting wild-type sperm-derived total or small RNAs into embryos [4]. This suggests that sperm-borne miRNAs are crucial for preimplantation embryonic development. Sperm-borne miRNAs modulate early embryonic development in mice [8], cows [9, 10], rabbits and humans [11–14]. For example, *miR-34c* is a member of the miR-34 family that is preferentially expressed in male gonads and functionally required for spermatogenesis [15, 16]. Low levels of *miR-34c* expression in semen and spermatozoa are associated with male subfertility [17–19]. The role of sperm-borne *miR-34c* in the first cleavage division of murine embryos remains unclear [8, 20], but an increasing number of studies have shown that *miR-34c* participates in early embryonic development. For example, in rabbits and cows, *miR-34c* improves the developmental competence of embryos generated by somatic cell nuclear transfer (SCNT) [10, 21]. In addition, we have demonstrated that spermatozoan *miR-34c* levels are correlated with intracytoplasmic sperm injection (ICSI) outcomes, such as embryo quality and clinical pregnancy and live birth rates [11]. Our results are supported by a recent study showing that *miR-34c* expression levels in sperm are associated with embryonic development kinetics and clinical pregnancy [13]. However, the mechanisms underlying the regulation of embryonic development by *miR-34c* are unknown.

In mammalian oocytes, maternal mRNA stored during oocyte growth supports oocyte maturation and early embryonic development. The zygotic genome then initiates transcription and controls subsequent embryonic development. Two crucial processes occur during the maternal-to-zygotic transition (MZT): degradation of maternal mRNA transcripts and activation of the zygotic genome [22]. Impaired maternal mRNA degradation prevents appropriate zygotic genome activation and arrests embryonic development [23], and the decay of maternal mRNA transcripts may be facilitated by the activity of miRNAs [24]. Although some studies have examined the possible involvement of sperm-borne miRNAs in regulating the homeostasis of zygotic transcriptomes [4, 9], to the best of our knowledge no studies have examined the involvement of sperm-borne miRNAs in the degradation of maternal mRNA.

Accordingly, in the current study, we explored the role of sperm-borne *miR-34c* in early embryonic development by monitoring the development of embryos microinjected with an *miR-34c* inhibitor or a negative-control RNA (NC) at the zygote stage. The mRNA expression profiles of the microinjected embryos were then determined by RNA sequencing (RNA-seq) analysis.

## Materials and methods

### Collection of mouse embryos

Female C57BL/6 mice were obtained from Shanghai Laboratory Animal Co., Ltd. (Shanghai, China). Experimental protocols involving mice were approved by the Zhejiang University Institutional Animal Care and Research Committee (Approval # ZJU20160202), and mouse care and use were in accordance with the relevant guidelines and regulations.

The mice (6–8 weeks old) were superovulated by intraperitoneal injection with 5 IU of pregnant mare serum gonadotropin (Sigma Aldrich, St. Louis, MO, USA), followed 48 h later by injection with 5 IU of human chorionic gonadotropin (hCG, Sigma). They were then mated with C57BL/6 male mice at a ratio of 1:1. Pronucleated zygotes were harvested 20–22 h after hCG injection, microinjected with an *miR-34c* inhibitor or a negative-control (NC) RNA and cultured in EmbryoMax Advanced KSOM Embryo Medium (Millipore, Burlington, MA, USA) under 5% CO_2_ in a humidified atmosphere at 37 °C. Embryos at the two-cell, four-cell and blastocyst stages were collected 44–50, 62–64 and 88–92 h after hCG injection, respectively.

### Microinjection of zygotes

Fertilized zygotes were collected 20–22 h after hCG injection. The zygotes of mice were microinjected with a customized miRCURY LNA miRNA Power Inhibitor targeting *miR-34c* (Qiagen, Hilden, Germany). To ensure the specificity of the experimental results, other zygotes of mice were microinjected with miRCURY LNA miRNA Inhibitor Negative Control (Qiagen, Hilden, Germany) as a control experiment. The microinjection of zygotes was performed as previously described [8]: approximately 10 pL of 20 µM *miR-34c* inhibitor or NC RNA were microinjected into the cytoplasm of a zygote using a micromanipulator (Eppendorf, Hamburg, Germany).

### Extraction of total RNA and whole-transcriptome amplification

Embryos at the same developmental stage (two-cell, four-cell or blastocyst stage) were pooled and randomly divided into *miR-34c* inhibitor and NC groups. Five embryos per group were collected in phosphate-buffered saline and stored at -80 °C. Embryonic transcriptome amplification was performed using a REPLI-g WTA Single Cell Kit (Qiagen), following the manufacturer’s instructions. The completion of lysis was confirmed by microscopy. The main steps were cell lysis, generation of complementary DNA, ligation and whole-transcriptome amplification. Amplified mRNAs and other RNAs with poly-adenine + tails were used for next-generation RNA-seq and reverse transcription–quantitative polymerase chain reaction (RT-qPCR).

### RNA-seq analysis

Paired-end libraries were synthesized using the TruSeq™ RNA Sample Preparation Kit (Illumina, San Diego, CA, USA), following the manufacturer’s instructions. Purified libraries were quantified using a Qubit® 2.0 Fluorometer (Life Technologies, Carlsbad, CA, USA). A 2100 Bioanalyzer (Agilent Technologies, Santa Clara, CA, USA) was used to assess RNA quality and quantity. RNA-seq was performed using a NovaSeq 6000 instrument (Illumina). Library construction and sequencing were performed by Shanghai Sinomics Corporation (Shanghai, China).

### Quantitative PCR

miRNA qPCR was performed using a miScript SYBR Green PCR kit (Qiagen), as we previously described [11]. miScript primer assays for *miR-34c* were performed on an ABI 7900HT real-time PCR system (Applied Biosystems, Waltham, MA, USA). The small nuclear RNAs *Rnu6B* and *Gapdh* were used for normalization. The primer sequences are listed in Table S1 in the Supplementary Files. All procedures were performed according to the manufacturers’ instructions. A quantification cycle (Cq) value < 37 was used as the cut-off, and the 2^−ΔΔ^Cq method was used to calculate relative expression levels of miRNA and mRNA. All experiments were performed in triplicate.

### Bioinformatics analysis

Cluster analysis and heat map visualization of differentially expressed mRNAs were performed using TBtools, which integrates various biological data-handling tools V1.082 [25]. Pathway and process enrichment were analyzed using Metascape software (https://metascape.org/) with the following ontology resources: Kyoto Encyclopedia of Genes and Genomes (KEGG) Pathways, Gene Ontology (GO) Biological Processes, Reactome Gene Sets, CORUM, Transcriptional Regulatory Relationships Unravelled by Sentence-Based Text-Mining (TRRUST), Pattern Gene Database (PaGenBase) and WikiPathways. RNAs with an enrichment factor greater than 1.5 were collected and grouped into clusters based on their similarities, where the enrichment factor is the ratio of the observed counts to the counts expected by chance. *P* < 0.05 was considered to indicate a statistically significant difference. To systematically analyze the biological functions, the different mRNAs were mapped using the STRING database (STRING, V11.0; https://string-db.org/), which predicts protein functional associations.

### Statistical analysis

Differences between two groups were evaluated using Student’s *t*-test or a nonparametric Mann–Whitney *U* test. Embryonic developmental outcomes were compared using a chi-square (χ^2^) test. Data are presented as means ± standard errors of the mean and were considered significantly different if *P* < 0.05. All data were analyzed using SigmaPlot version 14.0 (Systat Software Inc., Chicago, IL, USA).

## Results

### **Expression pattern of*miR-34c*in mouse preimplantation embryos**

**Fig. 1:**
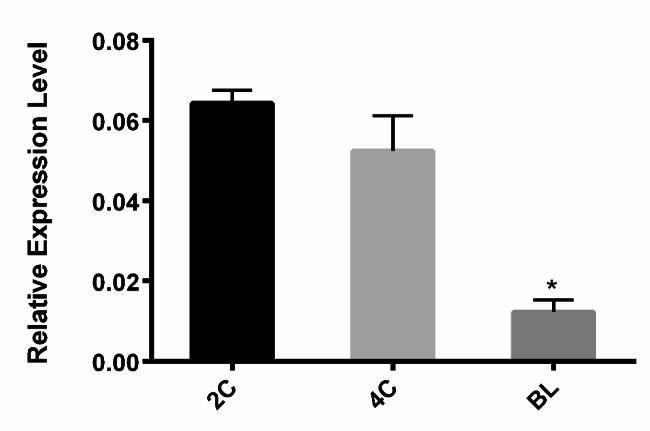
Level of *miR-34c* in mouse preimplantation embryos Reverse transcription–quantitative polymerase chain reaction was used to analyze the level of *miR-34c* in two-cell and four-cell stage embryos and blastocysts. Data are presented as means ± standard errors of the mean (*n* = 3). All values were calculated against quantification cycle values. Data were normalized to endogenous RNU6B expression. *: *P* < 0.01 compared with other groups

*miR-34c* is present in sperm but absent from oocytes, and zygotic *miR-34c* is derived from sperm [8]. Thus, we examined the levels of *miRNA-34c* in preimplantation mouse embryos using RT-qPCR (Fig. 1). *miR-34c* levels were significantly lower in blastocyst-stage embryos than in two-cell and four-cell embryos (*P* = 0.003 and *P* = 0.012, respectively). However, there was no significant difference between the levels of *miR-34c* in two-cell embryos and those in four-cell embryos (*P* = 0.270).

### **Microinjection of zygotes with an*miR-34c*inhibitor compromised embryonic development**

In the NC group, 76.2% of zygotes formed two-cell embryos, whereas in the *miR-34c* inhibitor group, only 62.0% of zygotes formed two-cell embryos (*P* = 0.003, χ^2^ = 9.08, Table 1). Similarly, a higher percentage of two-cell embryos developed to the four-cell stage in the NC group than in the *miR-34c* inhibitor group (*P* = 0.039, χ^2^ = 4.26). Moreover, the blastocyst formation rate was higher in the NC group than the *miR-34c* inhibitor group (40.8% vs. 23.2%, *P* = 0.002, χ^2^ = 9.43). These results indicate that sperm-borne *miR-34c* may play a role in preimplantation mouse embryonic development.

**Table 1:** Development of embryos injected with *miR-34c* inhibitor or NC in 2PN-stage.

	No. injected 2PN embryos	No. 2-cell (% of 2-cell/2PN)	No. 4-cell(%of 4-cell/2-cell)	No. blastocyst(% of blastocyst/2-cell)
NC group	193	147(76.2)	104(70.7)	60(40.8)
Inhibitor group	229	142(62.0)	83(58. 5)	33(23.2)
*P* value		0.003	0.039	0.002

*P* value use χ^2^test, compared to NC group

### **Microinjection of an*miR-34c*inhibitor caused altered gene expression in two-cell stage embryos**

Single-cell RNA-seq technology was used to perform whole-transcriptome amplification to identify changes in the levels of mRNAs responsible for the effect of sperm-borne *miR-34c* on preimplantation embryonic development. The Circos software package (http://www.circos.ca/software/) was used to map the reads obtained from RNA-seq for each sample to a specific position in the genome and display these in a circular graph that illustrates interactions (Fig. S1). We used the online software platform TargetScan (http://www.targetscan.org/) to predict the targets of *miR-34c*, which enabled us to identify *miR-34c* target genes that were dysregulated subsequent to microinjection with the *miR-34c* inhibitor. We identified 177 target genes (6-mer sites > 1) that were detected in both the NC and *miR-34c* inhibitor groups at the two-cell embryo stage.

**Fig. 2:**
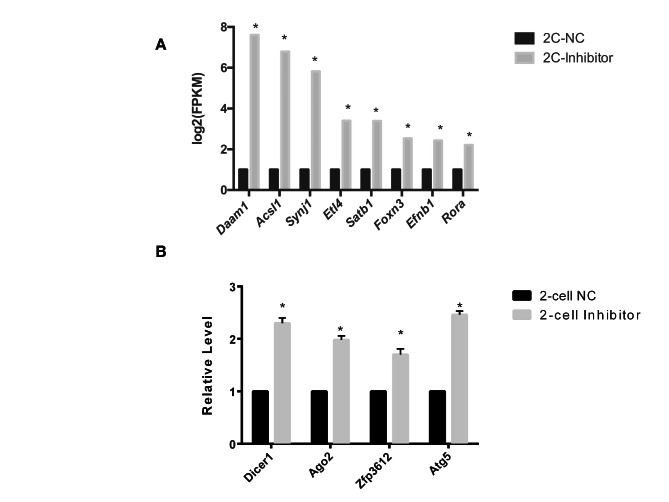
Levels of maternal messenger RNA (mRNA) expression in two-cell stage embryos microinjected with a *miR-34c* inhibitor or a negative control (NC) RNA. (**A**) Predicted *miR-34c* target maternal mRNA expression levels in two-cell stage embryos microinjected with an *miR-34c* inhibitor or the NC RNA. *: *P* < 0.05. (**B**) Four classical maternal mRNA expression levels in two-cell stage embryos microinjected with an *miR-34c* inhibitor or an NC RNA. *: *P* < 0.05. The 2^−ΔΔ^Cq method was used to calculate the relative expression levels

Maternal mRNA degradation is essential for MZT [26]. As zygotic genome activation (ZGA) occurs by the two-cell stage in mouse embryos, ZGA-dependent maternal mRNA degradation has been well characterized at this stage [26, 27]. We found that microinjection with the *miR-34c* inhibitor resulted in eight *miR-34c* target genes (*Daam1*, *Acsl1*, *Synj1*, *Etl4*, *Satb1*, *Foxn3*, *Efnb1* and *Rora*) being expressed at high levels at the two-cell stage. These eight genes encode maternal mRNAs that undergo metaphase II (MII)/two-cell degradation; i.e., their expression levels are high in MII oocytes, significantly decrease at the two-cell stage and then remain stable during the transition from the two-cell to the four-cell stage [27]. As shown in Fig. 2a, the expression levels of these *miR-34c*-targeted maternal mRNAs that undergo MII/two-cell degradation were higher in the *miR-34c* inhibitor group than in the NC group (*P* < 0.05). Furthermore, we evaluated the levels of four classical maternal genes that should be degraded before ZGA (*Dicer1*, *Ago2*, *Zfp3612* and *Atg5*) [28] in two-cell embryos. These four maternal mRNAs were present at higher levels in the *miR-34c* inhibitor group than in the NC group (all *P* < 0.05, Fig. 2b), suggesting that maternal degradation was affected by the *miR-34c* inhibitor.

### **Identification and enrichment analysis of differentially expressed genes in preimplantation embryos after microinjection of an*miR-34c*inhibitor**

**Fig. 3:**
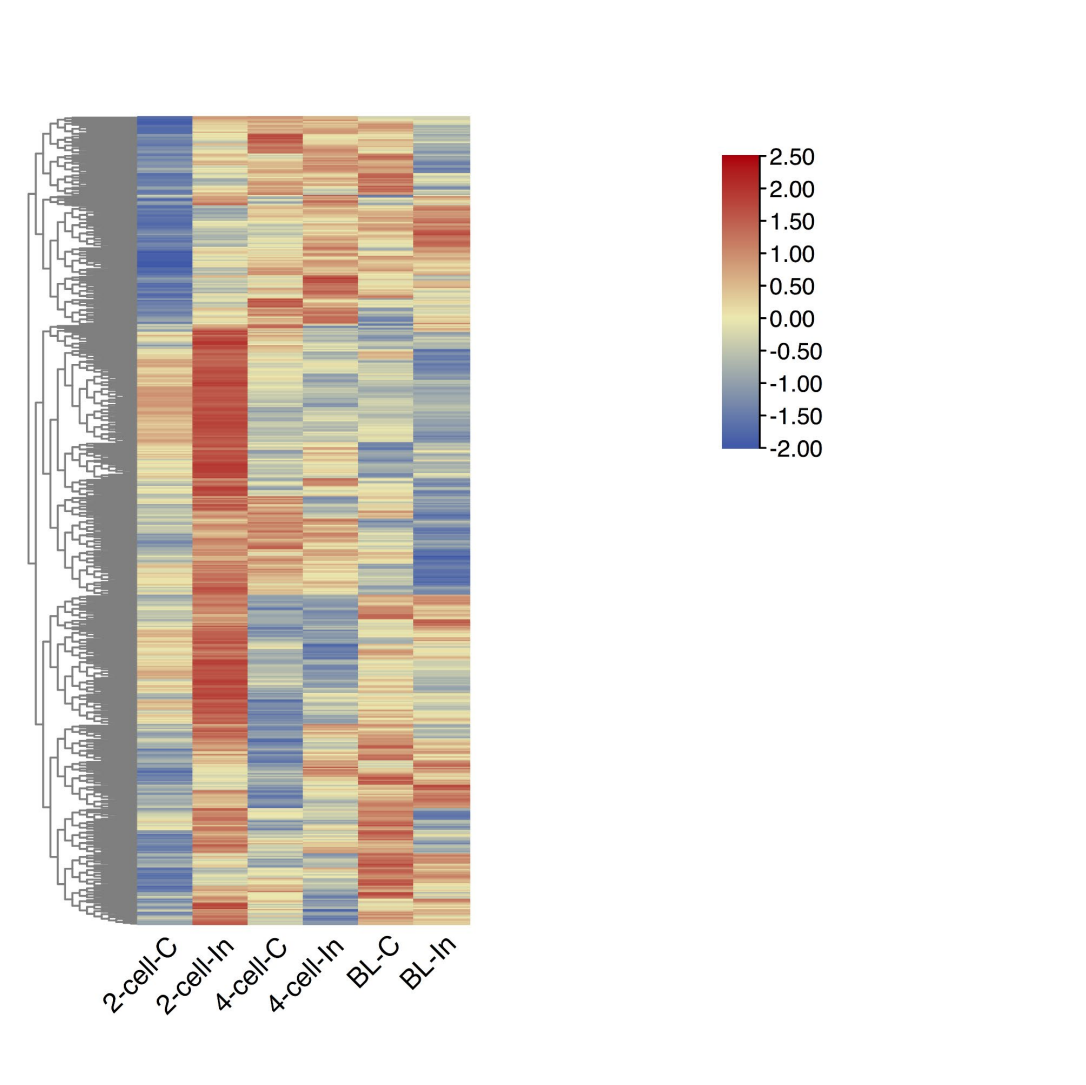
Transcriptomic profiling of mouse preimplantation embryos microinjected with an *miR-34c* inhibitor or an NC RNA. Heatmap of the differential expression levels of the messenger RNAs (mRNAs) in two-cell and four-cell stage embryos and blastocysts microinjected with an *miR-34c* inhibitor or an NC RNA. Columns represent different samples, and rows represent mRNAs. Dark red indicates high expression, and dark blue indicates low expression

We characterized the significantly dysregulated differentially expressed genes (DEGs) that were transcribed into mRNAs after microinjection of an *miR-34c* inhibitor (in comparison to their regulation in the NC group). We employed the Benjamini–Hochberg method to control the false discovery rate, with an adjusted *P*-value < 0.05 and log2 fold change (FC) > 1.00 as the thresholds. This revealed that 1,086 mRNAs were significantly dysregulated (624 upregulated and 462 downregulated) in two-cell embryos after microinjection of an *miR-34c* inhibitor. DEGs at the four-cell and blastocyst stages were also identified (Fig. 3).

We used |log2 FC| > 5.00 as a threshold to filter the DEGs and then investigated their functions. Pathway and process enrichment analyses of the DEGs were performed using the following online databases: KEGG Pathway, GO Biological Process, Reactome gene sets, CORUM, TRRUST, PaGenBase and WikiPathways. All of the genes in the *Mus musculus* Reference Sequence Genome (University of California Santa Cruz version: mm10) were used as the enrichment background. Terms with a *P*-value < 0.01, a minimum count of 3 and an enrichment factor > 1.5 were collected and grouped into clusters based on their similarities.

**Fig. 4:**
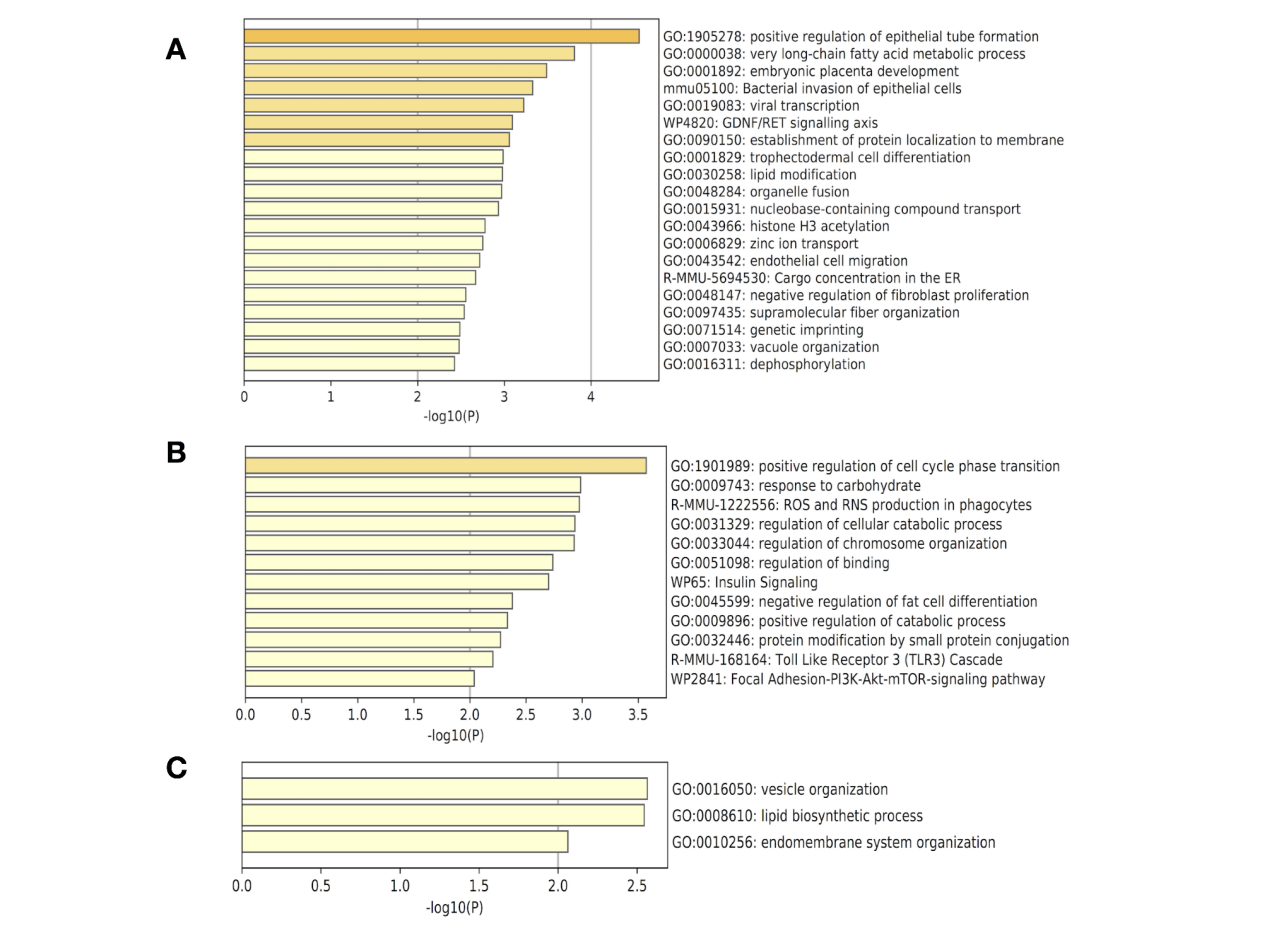
Enrichment analysis-based functional assignments of differentially expressed genes (DEGs) in preimplantation embryos (**A**) Bar graph of the most common biological function-associations of DEGs in two-cell stage embryos. (**B**) Bar graph of the most common biological function-associations of DEGs in four-cell stage embryos. (**C**) Bar graph of the most common biological function-associations of DEGs in blastocysts. Colored by *P*-values

In two-cell embryos, 103 of 177 DEGs were upregulated and the remaining 74 DEGs were downregulated after microinjection of the *miR-34c* inhibitor. The enrichment databases revealed that these DEGs were mainly involved in lipid metabolism and cellular membrane functions, such as very long-chain fatty acid metabolic processes (GO:0000038), nucleobase-containing compound transport (GO:0015931), protein localization to membranes (GO:0090150) and lipid modification (GO003025; Fig. 4a). In four-cell embryos, 64 of 121 DEGs were upregulated and the remaining 57 DEGs were downregulated. These DEGs were mainly involved in the regulation of cell-cycle phase transitions (GO:1,901,989), responses to carbohydrates (GO:0009743) and the regulation of cellular catabolic processes (GO:0031329; Fig. 4b), suggesting that the *miR-34c* inhibitor may have affected cell proliferation and energy metabolism in four-cell embryos. At the blastocyst stage, 17 of 24 DEGs were upregulated and the remaining seven DEGs were downregulated. These DEGs were involved in vesicle organization (GO:0016050), lipid biosynthetic process (GO:0008610) and endomembrane system organization (GO:0010256; Fig. 4c), suggesting that the *miR-34c* inhibitor may have affected the endomembrane system in blastocysts, including their intracellular organelles and secretory vesicles.

### **Altered transcriptome profiles in preimplantation embryos after*miR-34c*inhibitor microinjection**

To investigate earlier events in embryos at the two-cell, four-cell and blastocyst stages, we selected four DEGs that were most significantly downregulated from each group and confirmed their downregulation using RT-qPCR.

**Fig. 5:**
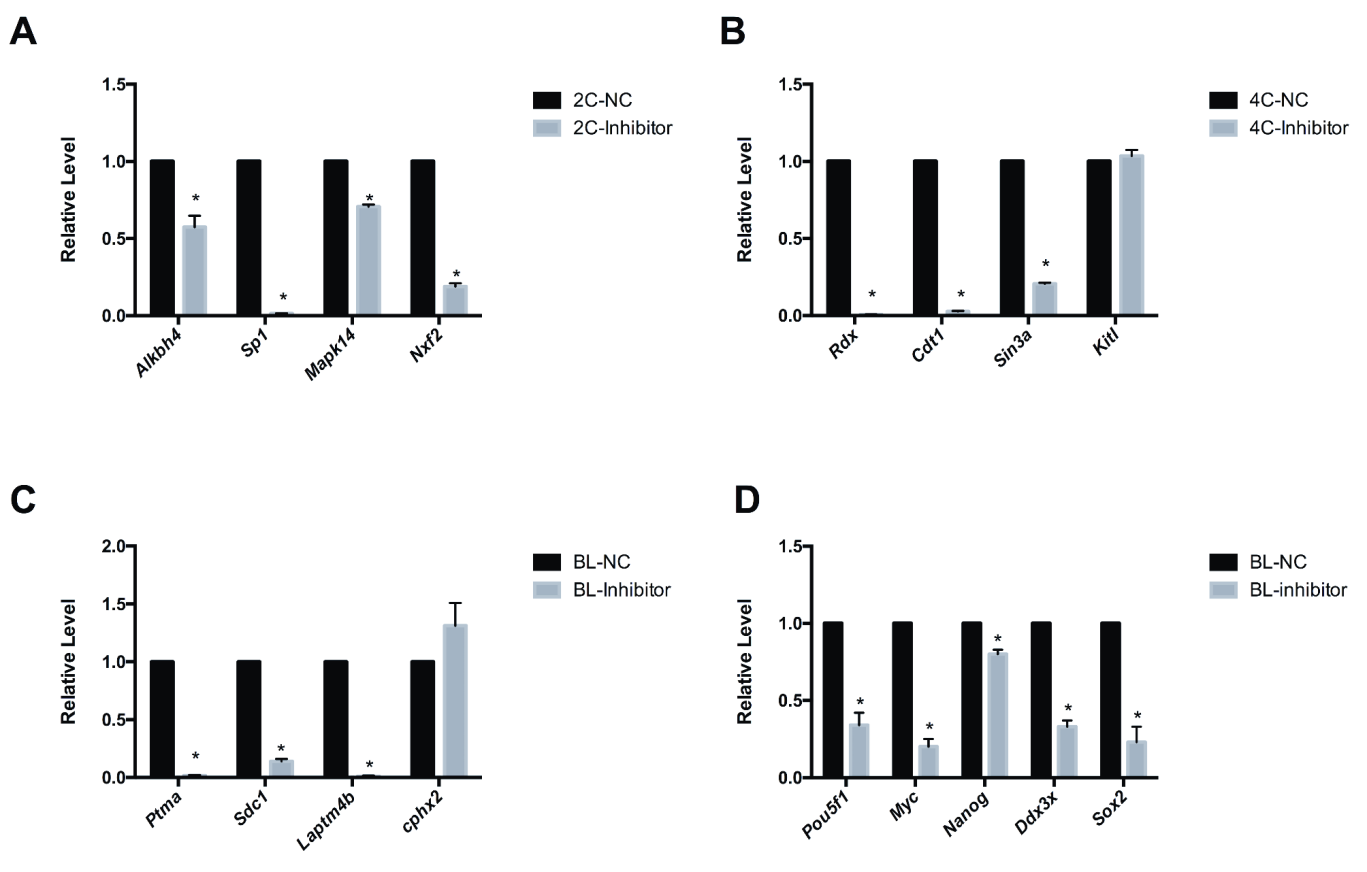
Expression levels of significantly dysregulated messenger RNAs and totipotency markers in preimplantation embryos (**A**) Expression levels of the four most dysregulated genes in two-cell stage embryos. (**B**) Expression levels of the four dysregulated genes in four-cell stage embryos. (**C**) Expression levels of the four most dysregulated genes in blastocysts. (**D**) Expression levels of classical totipotency markers in blastocysts. Data are presented as means ± standard errors of the mean (*n* = 3)

At the two-cell stage, all four of the most significantly downregulated DEGs (*Alkbh4*, *Sp1*, *Mapk14* and *Nxf2*) were confirmed to be downregulated after *miR-34c* inhibitor microinjection (Fig. 5a). In four-cell embryos, three of the four most significantly downregulated DEGs (*Rdx*, *Cdt1* and *Sin3a*) were confirmed to be downregulated after microinjection of the *miR-34c* inhibitor (Fig. 5b). However, *Kitl* was observed to be unaltered when tested by RT-qPCR. Furthermore, we demonstrated that the expression of *Ptma*, *Sdc-1* and *Laptm4b* was downregulated in blastocysts derived from the zygotes microinjected with the *miR-34c* inhibitor, whereas *Cphx2* expression did not differ significantly between the *miR-34c* inhibitor and NC groups (Fig. 5c). We also evaluated the mRNA levels of classical embryo-specific activation and totipotency markers (*Pou5f1*, *Myc*, *Nanog*, *Ddx3x* and *Sox2*) [29] in blastocysts. As shown in Fig. 5d, the expression of these markers was significantly downregulated after microinjection of zygotes with the *miR-34c* inhibitor (all *P* < 0.05), indicating the negative effect of *miR-34c* inhibition on the totipotency of preimplantation mouse embryos.

## Discussion

Embryos derived from ICSI using spermatozoa with aberrant miRNA profiles display reduced preimplantation developmental potential and aberrant gene expression profiles, indicating that sperm-borne miRNAs are crucial for preimplantation embryonic development [4]. The roles of sperm-borne *miR-34c* in the first cleavage division of mouse embryos [8] [20] and outcomes of human IVF [11, 13] have been explored, but the mechanisms whereby sperm-borne *miR-34c* regulates preimplantation embryonic development remain to be elucidated. In the current study, we found that compared with controls, zygotes microinjected with an *miR-34c* inhibitor had reduced developmental potential and altered gene expression profiles. Moreover, we determined that microinjection of an *miR-34c* inhibitor caused upregulation of the expression of maternal *miR-34c* target mRNAs and classical maternal mRNAs in two-cell embryos, suggesting that sperm-borne *miR-34c* may be involved in the degradation of maternal mRNA. To the best of our knowledge, this has not been previously reported.

Liu et al. found that more than 70% of zygotes microinjected with an *miR-34c* inhibitor failed to cleave, whereas less than 3% of zygotes microinjected with a scrambled inhibitor were arrested [8]. However, we found that the *miR-34c* inhibitor we used had only a mild effect on the first cleavage (76.2% vs. 62.0%). In our study, we used C57BL/6 mice, in contrast to the ICR mice used by Liu et al. [8], as well as a miRCURY LNA miRNA Power Inhibitor, which targets *miR-34c* with high efficiency and specificity, for microinjections. Although the choice of mouse strain should be considered first to explain the discrepancy between the results of these studies, the technical limitations of microinjection cannot be ruled out. Another study found that *miR-34b/c*-null sperm led to normal fertilization, preimplantation development and birth rates, showing that *miR-34b/c* is not essential for fertilization or preimplantation development [20]. These conflicting results may be caused by variations in experimental designs and methods and may also reflect the functional redundancy of miRNAs due to their polycistronic and paralogous features [30]. Moreover, mRNAs and miRNAs interact in a reciprocal one-to-multiple regulatory manner: a single miRNA can bind multiple mRNAs, and single mRNAs can be bound by different miRNAs [31]. Therefore, most loss-of-function studies of single miRNAs have only identified minor phenotypic changes [30]. However, the expression level of *miR-34c* in sperm is associated with clinical outcomes in both IVF [13] and ICSI cycles [11], and *miR-34c* expression in donor cells enhances the developmental potential of embryos generated by SCNT [10]. In the present study, blastocyst formation was significantly affected by *miR-34c* inhibitor microinjection at the zygote stage. Thus, we cannot exclude the possibility that sperm-borne *miR-34c* affects preimplantation embryonic development.

MZT is the earliest step in animal embryogenesis and comprises two processes: the degradation of maternal RNAs and the initiation of transcription of the embryonic genome. In mouse embryos, 90% of maternally stored RNAs are degraded by the two-cell stage [32]. When the decay of maternal mRNA is impaired in embryos derived from *Btg4-*null female mice, zygotes arrest at the one- to two-cell stage [4]. In addition, the degradation of maternal mRNA is essential for both mouse and human embryonic development [26]. It was suggested that miRNAs participate in the decay of maternal transcripts by blocking translation and inducing the destabilization of hundreds of different maternal targets [24]. However, the involvement of sperm-borne miRNAs in maternal mRNA degradation has not been reported. We found that compared with controls, microinjection of the *miR-34c* inhibitor increased the expression levels of maternal *miR-34c* target mRNAs and classical maternal genes in two-cell stage embryos, suggesting that *miR-34c* may influence preimplantation embryonic development by affecting the degradation of maternal mRNA. However, the results should be interpreted with caution: embryos from C57BL/6 mice could more likely develop to blastocysts in in vitro culture, whereas those from other strains would probably be arrested at the 2-cell stage (2-cell block). Due to the difference in the MZT process between blocking and non-blocking strains, the role of *miR-34c* in maternal mRNA degradation and preimplantation embryonic development of other strains, especially mice presenting 2-cell block, should be further investigated.

Several genes involved in preimplantation embryonic development, including *Alkbh4*, *Sp1*, *Mapk14* and *Sin3a*, were significantly downregulated in two-cell and four-cell embryos derived from zygotes microinjected after microinjection of the *miR-34c* inhibitor. AlkB homolog4 (A*lkbh4*) modulates fundamental processes, including cytokinesis and cell motility, and its depletion is lethal during the early preimplantation embryo stage [33]. Sp1 is present in preimplantation embryos [34], and its concentration increases gradually from the one-cell stage to the blastocyst stage [34, 35]. As Sp1 is a transcription factor, it is thought to be crucial for the transcription of genes involved in cell proliferation and differentiation, which are essential processes during early embryonic development. *Mapk14* mRNA is expressed throughout murine preimplantation development, and two-cell embryos cultured in the presence of a MAPK14 inhibitor arrest at the 8- to 16-cell stage [33]. These findings suggest that MAPK14 plays a distinct role in preimplantation embryos. Suppressor-interacting 3a (SIN3a) is a scaffold component of the chromatin repressive SIN3/histone deacetylase (HDAC) complex [36]. SIN3a is detectable throughout preimplantation development [37], and RNA-seq analysis has shown that *Sin3a* is a conserved hub gene of mouse and human transcriptomic networks in preimplantation embryos [38, 39]. Depletion of SIN3a impairs cell cycle progression, alters transcriptomic profiles and disrupts HDAC1 activity in mouse preimplantation embryos, indicating that SIN3a regulates the progression of embryonic development via HDAC1[39]. Our results show that the microinjection of zygotes with the *miR-34c* inhibitor downregulated the expression of genes related to embryonic development at the cleavage stage. As our bioinformatics analyses revealed that DEGs in the two- and four-cell stages were related to cell proliferation and cellular metabolism, we suggest that sperm-borne *miR-34c* may have a role in embryonic metabolism, which is crucial for cell proliferation, embryo developmental competence and epigenetic reprogramming [40–42].

We determined that DEGs enriched in blastocysts were related to the endomembrane system and vesicle organization. Eukaryotic cells are equipped with a set of interrelated endomembrane systems consisting of intracellular organelles and secretory extracellular vesicles (EVs). Blastocysts prepare for implantation by interacting with the maternal endometrium. Embryos cultured in vitro contain EVs with an average size of 100 nm, and in vivo and in vitro experiments have demonstrated that embryo-derived EVs are locally absorbed into the endometrial epithelium [43]. These results suggest that embryonic EVs may aid implantation by altering the epithelial physiology of the endometrium. We also demonstrated that *Sdc1* and *Laptm4b* were downregulated in blastocysts microinjected with the *miR-34c* inhibitor. Syndecan-1 (SDC1) is the co-receptor of CXCL1, a chemokine that plays a key role in embryonic implantation. SDC1 regulates CXCL1 expression and trophoblast invasion into the endometrium during the peri-implantation period [44]. As a member of the lysosomal-associated protein transmembrane (LAPTM) family, LAPTM4B affect membrane properties of EVs by regulating lipid composition [45]. Based on the above-described results of the current study and other studies, we suggest that sperm-borne *miR-34c* may be involved in the development of implantation-competent blastocysts.

## Conclusions

In summary, we found that preimplantation embryonic developmental potential was compromised by microinjection of zygotes with an *miR-34c* inhibitor. Moreover, we observed that this treatment resulted in the alteration of gene expression profiles, which suggests that sperm-borne *miR-34c* may modulate preimplantation embryonic development by influencing multiple biological processes. Our analyses revealed that these processes may include maternal mRNA degradation, cellular metabolism, cell proliferation and blastocyst implantation-competence. Our data illustrate the importance of sperm-derived miRNAs in the development of preimplantation embryos.

## Electronic supplementary material

Below is the link to the electronic supplementary material.

**Additional file 1: Fig. S1** A circular graph of RNA-sequencing (RNA-seq) reads of each sample, mapped to their position on the genome. S2-cell-In: Two-cell stage RNA-seq reads performed after microinjection of an *miR-34c* inhibitor, mapped to their position on the genome. S2-cell-C: Two-cell stage RNA-seq reads performed after microinjection of a negative control (NC) RNA, mapped to their position on the genome. S4-cell-In: Four-cell stage RNA-seq reads performed after microinjection of an *miR-34c* inhibitor, mapped to their position on the genome. S4-cell-C: Four-cell stage RNA-seq reads performed after microinjection of an NC RNA, mapped to their position on the genome. BL-In: Blastocyst stage RNA-seq reads performed after microinjection of an *miR-34c* inhibitor, mapped to their position on the genome. BL-C: Blastocyst stage RNA-seq reads performed after microinjection of an NC RNA, mapped to their position on the genome.

**Additional file 2: Table S1** Primer information.

## Data Availability

The original data presented in the study are included in the article. Further inquiries can be directed to the corresponding authors.

## Declarations

Experimental protocols involving mice were approved by the Zhejiang University Institutional Animal Care and Research Committee (Approval # ZJU20160202), and mouse care and use were in accordance with the relevant guidelines and regulations.

## Abbreviations

miRNA: microRNA
SCNT: somatic cell nuclear transfer
ICSI: intracytoplasmic sperm injection
MZT: maternal-to-zygotic transition
RNA-seq: RNA sequencing
DEGs: differentially expressed genes
IVF: in vitro fertilization
SIN3a: Suppressor interacting 3a
EV: extracellular vesicles
LAPTM: lysosomal-associated protein transmembrane
EGFR: epidermal growth factor receptor
