# Supplementary figures and images for: Sperm-borne microRNA-34c regulates maternal mRNA degradation and preimplantation embryonic development in mice
Source: Reprod Biol Endocrinol. 2023 Apr 26;21:40. doi: 10.1186/s12958-023-01089-3 (PMC10131327; doi:10.1186/s12958-023-01089-3)

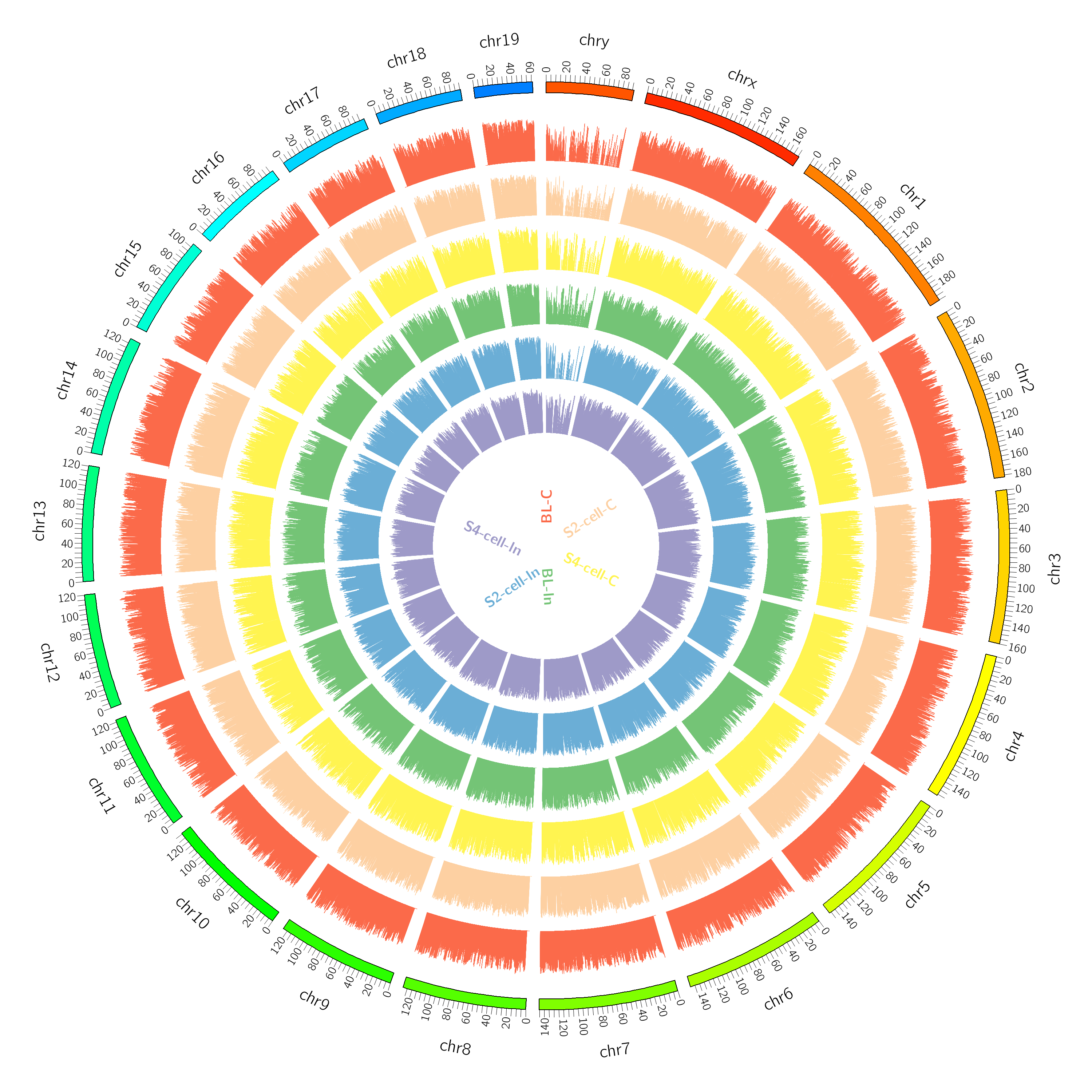

Supplement: Supplementary file 1 — Additional file 1: Fig. S1 A circular graph of RNA-sequencing (RNA-seq) reads of each sample, mapped to their position on the genome. S2-cell-In: Two-cell stage RNA-seq reads performed after microinjection of an miR-34c inhibitor, mapped to their position on the genome. S2-cell-C: Two-cell stage RNA-seq reads performed after microinjection of a negative control (NC) RNA, mapped to their position on the genome. S4-cell-In: Four-cell stage RNA-seq reads performed after microinjection of an miR-34c inhibitor, mapped to their position on the genome. S4-cell-C: Four-cell stage RNA-seq reads performed after microinjection of an NC RNA, mapped to their position on the genome. BL-In: Blastocyst stage RNA-seq reads performed after microinjection of an miR-34c inhibitor, mapped to their position on the genome. BL-C: Blastocyst stage RNA-seq reads performed after microinjection of an NC RNA, mapped to their position on the genome. [file 12958_2023_1089_MOESM1_ESM.tiff]
