# Supplementary material for: Sperm-borne microRNA-34c regulates maternal mRNA degradation and preimplantation embryonic development in mice
Source: Reprod Biol Endocrinol. 2023 Apr 26;21:40. doi: 10.1186/s12958-023-01089-3 (PMC10131327; doi:10.1186/s12958-023-01089-3)
Supplement: Supplementary file 2 — Additional file 2: Table S1 Primer information. [file 12958_2023_1089_MOESM2_ESM.docx]

Supplementary. Table.1 Primer information

| **Primer** | **(5'to3')** |
| --- | --- |
| *Alkbh4-f* | CCTTGGACTCTTGCCTTTTCTA |
| *Alkbh4-r* | TCCCTCCACATATGACACTCAG |
| *Sp1-f* | CCACTCCTTCAGCCCTTATT |
| *Sp1-r* | CTCTCTCTTCCAGATGTCTC |
| *Mapk14-f* | CCGAACGATACCAGAACCTGTC |
| *Mapk-r* | ACGCAACTCTCGGTAGGTCCTT |
| *Nxf2-f* | CTCTGTGCAGTCTGGAATGAAA |
| *Nxf2-r* | TTGGTGTAGTCCCACTCG |
| *Radixin -f* | CTCTTACCACAGCGTGTTTTGG |
| *Radixin -r* | TTCCCTTAGCATCCCTCTGTGT |
| *Cdt1-f* | TGTTCAGGAGATGATGCGCA |
| *Cdt1-r* | TGATGCTGTCCTTGAAGGTGG |
| *Sin3a-f* | GCTCCTGGACACAGAAGAGG |
| *Sin3a-r* | TGTGCCAGATGTTCTCGAAG |
| *Kitl-f* | GAGCTCCAGAACAGCTAAACG |
| *Kitl-r* | CACTCCACAAGGTCATCCAC |
| *Prothymosin-a-f* | AAGGAGAAGAAGGAAGTTGTGGA |
| *Prothymosin-a-r* | CTACCTCATTGTCAGCCTCCTG |
| *Syndecan 1-f* | TGTTGCCAGATCACATGGCT |
| *Syndecan 1-r* | GGCCAGTTAGGCTCCCATTC |
| *Laptm4b-f* | CACCTTACGTGTCCGCCT |
| *Laptm4b-r* | GCAAGGACCAAGCAGGAG |
| *Cphx2-f* | GAGTTTCGACATGTCTTCCCAAG |
| *Cphx2-r* | TGACCTGTGGCTATGGTTCTGT |
| *18s-f* | GTAACCCGTTGAACCCCATT |
| *18s-r* | CCATCCAATCGGTAGTAGCG |
| *Gapdh-f* | GACCTCAACTACATGGTCTACA |
| *Gapdh-r* | ACTCCACGACATACTCAGCAC |
| *Pou5f1-f* | TGAGCCGTCTTTCCACCAGGC |
| *Pou5f1-r* | GGAAGCTTAGCCAGGTTCGAGGATC |
| *Nanog-f* | TTTATTGGTGCCAGAGCAAACC |
| *Nanog-r* | GTCTCCAAAGCCTAGAGTTAAC |
| *Sox2-f* | CCCACCTACAGCATGTCCTAC |
| *Sox2-r* | GCCTCGGACTTGACCACAG |
| *Myc-f* | TCGCTGCTGTCCTCCGAGTCC |
| *Myc-r* | GGTTTGCCTCTTCTCCACAGAC |
| *Ddx3x -f* | CTATGCCTCCAAAAGGTGTCCG |
| *Ddx3x -r* | AGACCCAACTCTTCCTACAGCC |
